# *N*-(2-Chloro­phenyl­sulfon­yl)acetamide

**DOI:** 10.1107/S1600536811012785

**Published:** 2011-04-13

**Authors:** K. Shakuntala, Sabine Foro, B. Thimme Gowda

**Affiliations:** a Department of Chemistry, Mangalore University, Mangalagangotri 574 199, Mangalore, India; b Institute of Materials Science, Darmstadt University of Technology, Petersenstrasse 23, D-64287 Darmstadt, Germany

## Abstract

The asymmetric unit of the title compound, C_8_H_8_ClNO_3_S, contains two independent mol­ecules in which the C—S—N—C torsion angles are −71.7 (3) and 61.2 (3)°. The benzene rings and the SO_2_—NH—CO—C segments form dihedral angles of 80.2 (1) and 88.1 (2)° in the two independent mol­ecules. In the crystal, inter­molecular N—H⋯O hydrogen bonds link the mol­ecules into chains in the *b*-axis direction.

## Related literature

For the sulfanilamide moiety in sulfonamide drugs, see; Maren (1976 ▶). For its ability to form hydrogen bonds in the solid state, see; Yang & Guillory (1972 ▶). For hydrogen-bonding modes of sulfonamides, see; Adsmond & Grant (2001 ▶). For our study of the effect of substituents on the structures of *N*-(ar­yl)-amides, see: Gowda *et al.* (2000 ▶), of *N*-(ar­yl)-methane­sulfonamides, see: Gowda *et al.* (2007 ▶) and of *N*-(substituted phenyl­sulfon­yl)-substituted amides, see: Gowda *et al.* (2010 ▶).
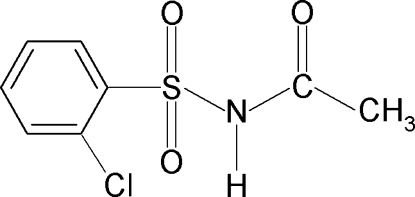


## Experimental

### 

#### Crystal data

C_8_H_8_ClNO_3_S
*M* *_r_* = 233.66
Monoclinic, 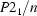

*a* = 11.215 (2) Å
*b* = 9.393 (2) Å
*c* = 19.655 (3) Å
β = 98.61 (2)°
*V* = 2047.2 (6) Å^3^
*Z* = 8
Mo *K*α radiation
μ = 0.56 mm^−1^
*T* = 293 K
0.16 × 0.16 × 0.04 mm

#### Data collection

Oxford Diffraction Xcalibur diffractometer with Sapphire CCD detector
Absorption correction: multi-scan (*CrysAlis RED*; Oxford Diffraction, 2009 ▶) *T*_min_ = 0.916, *T*_max_ = 0.978
8327 measured reflections
4168 independent reflections
1942 reflections with *I* > 2σ(*I*)
*R*_int_ = 0.048

#### Refinement

*R*[*F* ^2^ > 2σ(*F* ^2^)] = 0.057
*wR*(*F* ^2^) = 0.099
*S* = 0.95
4168 reflections
261 parameters
2 restraints
H atoms treated by a mixture of independent and constrained refinement
Δρ_max_ = 0.26 e Å^−3^
Δρ_min_ = −0.26 e Å^−3^

### 

Data collection: *CrysAlis CCD* (Oxford Diffraction, 2009 ▶); cell refinement: *CrysAlis RED* (Oxford Diffraction, 2009 ▶); data reduction: *CrysAlis RED*; program(s) used to solve structure: *SHELXS97* (Sheldrick, 2008 ▶); program(s) used to refine structure: *SHELXL97* (Sheldrick, 2008 ▶); molecular graphics: *PLATON* (Spek, 2009 ▶); software used to prepare material for publication: *SHELXL97*.

## Supplementary Material

Crystal structure: contains datablocks I, global. DOI: 10.1107/S1600536811012785/vm2086sup1.cif

Structure factors: contains datablocks I. DOI: 10.1107/S1600536811012785/vm2086Isup2.hkl

Additional supplementary materials:  crystallographic information; 3D view; checkCIF report

## Figures and Tables

**Table 1:** Hydrogen-bond geometry (Å, °)

*D*—H⋯*A*	*D*—H	H⋯*A*	*D*⋯*A*	*D*—H⋯*A*
N1—H1*N*⋯O3^i^	0.85 (2)	2.03 (2)	2.848 (4)	162 (3)
N2—H2*N*⋯O6^ii^	0.84 (2)	1.96 (2)	2.788 (4)	172 (3)

Symmetry codes: (i) 
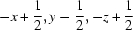
; (ii) 
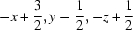
.

**Fig. 1.**
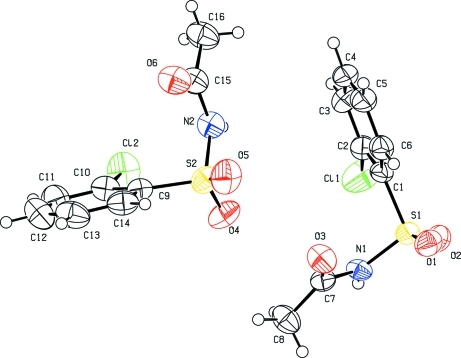
Molecular structure of the title compound, showing the atom- labelling scheme. Displacement ellipsoids are drawn at the 50% probability level.

**Fig. 2.**
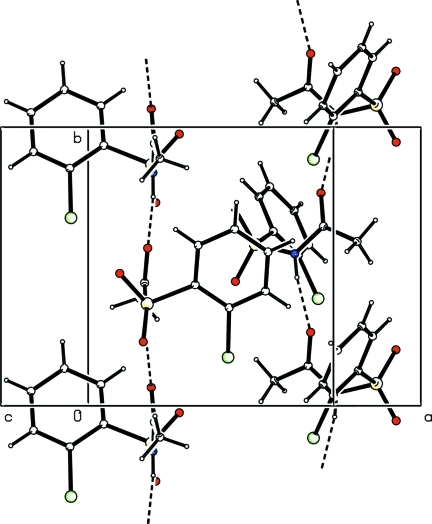
Molecular packing in the title compound. Hydrogen bonds are shown as dashed lines.

## supplementary crystallographic information

### Comment

The structures of sulfonamide drugs contain the sulfanilamide moiety (Maren, 1976). The affinity for hydrogen bonding in the solid state due to the presence of various hydrogen bond donors and acceptors give rise to polymorphism (Yang & Guillory, 1972). The hydrogen bonding preferences of sulfonamides have also been investigated (Adsmond & Grant, 2001). The nature and position of substituents play a significant role on the crystal structures of *N*-(aryl)-amides and *N*-(aryl)- sulfonoamides (Gowda *et al.*, 2000, 2007, 2010). As a part of studying the effects of substituents on the structures of this class of compounds, the structure of *N*-(2-chlorophenylsulfonyl)-acetamide (I) has been determined (Fig. 1). The asymmetric unit of (I) contains two independent molecules. The rms deviation of a fit of the inverted molecule 2 (containing Cl2) on molecule 1 (containing Cl1) is 0.278 Å for 12 fitted atoms (excluding H atoms and O atoms SO_2_ groups). In one of the molecules, the conformation of the N—C bond in the C—SO_2_—NH—C(O) segment has *gauche* torsions with respect to the S═O bonds, the torsional angles being C15—N2—S2—O5 = -54.0 (4)° and C15—N2—S2—O4 = 176.4 (3)°. The conformations of the N—H and C=O bonds of these segments are *anti* to each other, similar to that observed in *N*-(phenylsulfonyl)-acetamide (II) (Gowda *et al.*, 2010).

The molecules in (I) are bent at the *S*-atom with a C—S—N—C torsion angles of -71.7 (3)° and 61.2 (3)° in the two independent molecules, compared to the values of -58.8 (4)° in (II),

Further, the dihedral angles between the benzene rings and the SO_2_—NH—CO—C groups in (I) are 80.2 (1)° in molecule 1 and 88.1 (2)° in molecule 2, compared to the values of 89.0 (2)° in (II).

In the crystal structure, the intermolecular N–H···O hydrogen bonds (Table 1) link the molecules into chains in the *b*-direction. Part of the crystal structure is shown in Fig. 2.

### Experimental

The title compound was prepared by refluxing 2-chlorobenzenesulfonamide (0.10 mole) with an excess of acetyl chloride (0.20 mole) for one hour on a water bath. The reaction mixture was cooled and poured into ice cold water. The resulting solid was separated, washed thoroughly with water and dissolved in warm dilute sodium hydrogen carbonate solution. The title compound was reprecipitated by acidifying the filtered solution with glacial acetic acid. It was filtered, dried and recrystallized from ethanol. The purity of the compound was checked by determining its melting point. It was further characterized by recording its infrared spectra.

Plate like colourless single crystals of the title compound used in X-ray diffraction studies were obtained from a slow evaporation of an ethanolic solution of the compound.

### Refinement

The H atoms of the NH groups were located in a difference map and later restrained to the distance N—H = 0.86 (2) Å. The other H atoms were positioned with idealized geometry using a riding model with the aromatic C—H distance = 0.93 Å and methyl C—H = 0.96 Å. All H atoms were refined with isotropic displacement parameters (set to 1.2 times of the *U*_eq_ of the parent atom).

### Crystal data

**Table:**

C_8_H_8_ClNO_3_S	*F*(000) = 960
*M**_r_* = 233.66	*D*_x_ = 1.516 Mg m^−^^3^
Monoclinic, *P*2_1_/*n*	Mo *K*α radiation, λ = 0.71073 Å
Hall symbol: -P 2yn	Cell parameters from 1468 reflections
*a* = 11.215 (2) Å	θ = 2.6–27.9°
*b* = 9.393 (2) Å	µ = 0.56 mm^−^^1^
*c* = 19.655 (3) Å	*T* = 293 K
β = 98.61 (2)°	Plate, colourless
*V* = 2047.2 (6) Å^3^	0.16 × 0.16 × 0.04 mm
*Z* = 8	

### Data collection

**Table:**

Oxford Diffraction Xcalibur diffractometer with Sapphire CCD detector	4168 independent reflections
Radiation source: fine-focus sealed tube	1942 reflections with *I* > 2σ(*I*)
graphite	*R*_int_ = 0.048
Rotation method data acquisition using ω and φ scans	θ_max_ = 26.4°, θ_min_ = 2.9°
Absorption correction: multi-scan (*CrysAlis RED*; Oxford Diffraction, 2009)	*h* = −10→14
*T*_min_ = 0.916, *T*_max_ = 0.978	*k* = −9→11
8327 measured reflections	*l* = −24→19

### Refinement

**Table:**

Refinement on *F*^2^	Primary atom site location: structure-invariant direct methods
Least-squares matrix: full	Secondary atom site location: difference Fourier map
*R*[*F*^2^ > 2σ(*F*^2^)] = 0.057	Hydrogen site location: inferred from neighbouring sites
*wR*(*F*^2^) = 0.099	H atoms treated by a mixture of independent and constrained refinement
*S* = 0.95	*w* = 1/[σ^2^(*F*_o_^2^) + (0.0348*P*)^2^] where *P* = (*F*_o_^2^ + 2*F*_c_^2^)/3
4168 reflections	(Δ/σ)_max_ < 0.001
261 parameters	Δρ_max_ = 0.26 e Å^−^^3^
2 restraints	Δρ_min_ = −0.26 e Å^−^^3^

### Special details

**Table:**

Experimental. Absorption correction: CrysAlis RED (Oxford Diffraction, 2009) Empirical absorption correction using spherical harmonics, implemented in SCALE3 ABSPACK scaling algorithm.
Geometry. All e.s.d.'s (except the e.s.d. in the dihedral angle between two l.s. planes) are estimated using the full covariance matrix. The cell e.s.d.'s are taken into account individually in the estimation of e.s.d.'s in distances, angles and torsion angles; correlations between e.s.d.'s in cell parameters are only used when they are defined by crystal symmetry. An approximate (isotropic) treatment of cell e.s.d.'s is used for estimating e.s.d.'s involving l.s. planes.
Refinement. Refinement of *F*^2^ against ALL reflections. The weighted *R*-factor *wR* and goodness of fit *S* are based on *F*^2^, conventional *R*-factors *R* are based on *F*, with *F* set to zero for negative *F*^2^. The threshold expression of *F*^2^ > σ(*F*^2^) is used only for calculating *R*-factors(gt) *etc*. and is not relevant to the choice of reflections for refinement. *R*-factors based on *F*^2^ are statistically about twice as large as those based on *F*, and *R*- factors based on ALL data will be even larger.

### Fractional atomic coordinates and isotropic or equivalent isotropic displacement parameters (Å^2^)

**Table:**

	*x*	*y*	*z*	*U*_iso_*/*U*_eq_	
Cl1	0.51810 (9)	0.17437 (11)	0.37185 (7)	0.0865 (4)	
S1	0.27802 (8)	0.36595 (10)	0.38303 (5)	0.0430 (3)	
O1	0.19970 (19)	0.4751 (2)	0.39993 (13)	0.0544 (7)	
O2	0.27435 (19)	0.2294 (2)	0.41421 (12)	0.0536 (7)	
O3	0.2486 (2)	0.5660 (3)	0.26563 (13)	0.0541 (7)	
N1	0.2528 (2)	0.3364 (3)	0.29999 (16)	0.0379 (8)	
H1N	0.248 (3)	0.249 (2)	0.2887 (17)	0.045*	
C1	0.4262 (3)	0.4343 (4)	0.39817 (17)	0.0374 (9)	
C2	0.5274 (3)	0.3523 (4)	0.39431 (19)	0.0474 (10)	
C3	0.6403 (3)	0.4118 (5)	0.4089 (2)	0.0562 (11)	
H3	0.7082	0.3568	0.4057	0.067*	
C4	0.6532 (3)	0.5523 (5)	0.4280 (2)	0.0572 (11)	
H4	0.7298	0.5916	0.4388	0.069*	
C5	0.5534 (4)	0.6345 (4)	0.43133 (19)	0.0549 (11)	
H5	0.5623	0.7299	0.4437	0.066*	
C6	0.4403 (3)	0.5763 (4)	0.41646 (18)	0.0455 (10)	
H6	0.3727	0.6325	0.4187	0.055*	
C7	0.2354 (3)	0.4414 (4)	0.2506 (2)	0.0382 (9)	
C8	0.1998 (3)	0.3873 (4)	0.17924 (19)	0.0552 (11)	
H8A	0.2523	0.3105	0.1709	0.066*	
H8B	0.2062	0.4627	0.1470	0.066*	
H8C	0.1181	0.3537	0.1738	0.066*	
Cl2	0.71788 (9)	0.38447 (10)	0.10127 (6)	0.0742 (4)	
S2	0.55209 (8)	0.57945 (11)	0.18903 (6)	0.0530 (3)	
O4	0.4912 (2)	0.4460 (3)	0.18511 (14)	0.0686 (8)	
O5	0.5000 (2)	0.7009 (3)	0.21600 (15)	0.0696 (8)	
O6	0.7597 (2)	0.7639 (3)	0.22798 (14)	0.0606 (8)	
N2	0.6830 (3)	0.5457 (3)	0.23640 (17)	0.0460 (8)	
H2N	0.693 (3)	0.460 (2)	0.2471 (18)	0.055*	
C9	0.5848 (3)	0.6257 (4)	0.10696 (19)	0.0399 (9)	
C10	0.6495 (3)	0.5395 (4)	0.0678 (2)	0.0477 (10)	
C11	0.6630 (3)	0.5774 (5)	0.0015 (2)	0.0643 (12)	
H11	0.7069	0.5194	−0.0241	0.077*	
C12	0.6121 (4)	0.6996 (5)	−0.0266 (2)	0.0698 (13)	
H12	0.6203	0.7244	−0.0715	0.084*	
C13	0.5487 (3)	0.7862 (4)	0.0114 (3)	0.0652 (13)	
H13	0.5151	0.8702	−0.0077	0.078*	
C14	0.5342 (3)	0.7500 (4)	0.0776 (2)	0.0530 (11)	
H14	0.4903	0.8091	0.1026	0.064*	
C15	0.7731 (3)	0.6440 (4)	0.2501 (2)	0.0486 (10)	
C16	0.8857 (3)	0.5899 (4)	0.2931 (2)	0.0636 (12)	
H16A	0.8666	0.5536	0.3358	0.076*	
H16B	0.9429	0.6662	0.3021	0.076*	
H16C	0.9199	0.5151	0.2688	0.076*	

### Atomic displacement parameters (Å^2^)

**Table:**

	*U*^11^	*U*^22^	*U*^33^	*U*^12^	*U*^13^	*U*^23^
Cl1	0.0709 (7)	0.0570 (7)	0.1361 (13)	−0.0036 (6)	0.0295 (7)	−0.0294 (8)
S1	0.0420 (5)	0.0432 (6)	0.0434 (7)	−0.0090 (5)	0.0055 (4)	−0.0003 (5)
O1	0.0476 (15)	0.0558 (17)	0.0618 (19)	−0.0011 (12)	0.0151 (12)	−0.0133 (15)
O2	0.0601 (15)	0.0492 (16)	0.051 (2)	−0.0157 (12)	0.0075 (13)	0.0148 (14)
O3	0.0791 (17)	0.0272 (15)	0.054 (2)	−0.0058 (13)	0.0025 (13)	0.0003 (14)
N1	0.0472 (17)	0.0241 (16)	0.039 (2)	−0.0065 (14)	−0.0047 (14)	−0.0022 (17)
C1	0.040 (2)	0.037 (2)	0.034 (2)	−0.0069 (17)	0.0015 (16)	−0.0035 (19)
C2	0.048 (2)	0.049 (2)	0.046 (3)	−0.0083 (19)	0.0077 (18)	−0.005 (2)
C3	0.041 (2)	0.071 (3)	0.057 (3)	−0.004 (2)	0.0120 (18)	−0.002 (3)
C4	0.050 (3)	0.072 (3)	0.048 (3)	−0.028 (2)	0.0022 (19)	0.005 (3)
C5	0.062 (3)	0.053 (3)	0.048 (3)	−0.021 (2)	0.002 (2)	0.003 (2)
C6	0.048 (2)	0.045 (2)	0.042 (3)	−0.0023 (19)	0.0035 (17)	0.005 (2)
C7	0.038 (2)	0.031 (2)	0.045 (3)	−0.0024 (17)	0.0037 (17)	−0.001 (2)
C8	0.073 (3)	0.046 (3)	0.045 (3)	0.004 (2)	0.000 (2)	0.002 (2)
Cl2	0.0904 (8)	0.0504 (7)	0.0851 (10)	0.0276 (6)	0.0237 (6)	−0.0008 (6)
S2	0.0460 (6)	0.0515 (7)	0.0632 (8)	0.0038 (5)	0.0141 (5)	−0.0027 (6)
O4	0.0527 (15)	0.0630 (19)	0.091 (2)	−0.0193 (14)	0.0146 (14)	0.0033 (17)
O5	0.0653 (17)	0.0676 (19)	0.081 (2)	0.0259 (14)	0.0283 (15)	−0.0087 (17)
O6	0.0748 (18)	0.0335 (16)	0.069 (2)	−0.0025 (14)	−0.0023 (14)	−0.0024 (16)
N2	0.0552 (19)	0.0281 (17)	0.054 (2)	0.0019 (16)	0.0048 (16)	0.0010 (18)
C9	0.0354 (19)	0.037 (2)	0.046 (3)	0.0006 (17)	0.0034 (17)	−0.004 (2)
C10	0.047 (2)	0.039 (2)	0.055 (3)	0.0045 (18)	0.001 (2)	−0.003 (2)
C11	0.074 (3)	0.059 (3)	0.060 (4)	0.005 (2)	0.013 (2)	−0.004 (3)
C12	0.091 (3)	0.067 (3)	0.049 (3)	0.000 (3)	0.001 (3)	0.000 (3)
C13	0.069 (3)	0.051 (3)	0.068 (4)	0.006 (2)	−0.014 (3)	0.008 (3)
C14	0.044 (2)	0.046 (3)	0.066 (4)	0.0048 (19)	−0.001 (2)	−0.006 (3)
C15	0.056 (3)	0.039 (2)	0.050 (3)	0.003 (2)	0.005 (2)	−0.008 (2)
C16	0.070 (3)	0.054 (3)	0.061 (3)	0.004 (2)	−0.009 (2)	0.005 (2)

### Geometric parameters (Å, °)

**Table:**

Cl1—C2	1.728 (4)	Cl2—C10	1.729 (4)
S1—O1	1.421 (2)	S2—O5	1.420 (2)
S1—O2	1.425 (2)	S2—O4	1.424 (2)
S1—N1	1.638 (3)	S2—N2	1.647 (3)
S1—C1	1.765 (3)	S2—C9	1.761 (4)
O3—C7	1.210 (4)	O6—C15	1.209 (4)
N1—C7	1.377 (4)	N2—C15	1.366 (4)
N1—H1N	0.850 (17)	N2—H2N	0.835 (17)
C1—C2	1.383 (4)	C9—C14	1.386 (4)
C1—C6	1.384 (4)	C9—C10	1.393 (5)
C2—C3	1.375 (4)	C10—C11	1.382 (5)
C3—C4	1.375 (5)	C11—C12	1.361 (5)
C3—H3	0.9300	C11—H11	0.9300
C4—C5	1.369 (5)	C12—C13	1.373 (5)
C4—H4	0.9300	C12—H12	0.9300
C5—C6	1.373 (4)	C13—C14	1.377 (5)
C5—H5	0.9300	C13—H13	0.9300
C6—H6	0.9300	C14—H14	0.9300
C7—C8	1.490 (5)	C15—C16	1.499 (4)
C8—H8A	0.9600	C16—H16A	0.9600
C8—H8B	0.9600	C16—H16B	0.9600
C8—H8C	0.9600	C16—H16C	0.9600
			
O1—S1—O2	119.34 (15)	O5—S2—O4	120.45 (16)
O1—S1—N1	109.54 (15)	O5—S2—N2	108.97 (17)
O2—S1—N1	105.33 (15)	O4—S2—N2	103.94 (16)
O1—S1—C1	107.31 (16)	O5—S2—C9	107.31 (17)
O2—S1—C1	110.00 (15)	O4—S2—C9	109.35 (17)
N1—S1—C1	104.32 (15)	N2—S2—C9	105.95 (16)
C7—N1—S1	124.5 (2)	C15—N2—S2	123.5 (2)
C7—N1—H1N	121 (2)	C15—N2—H2N	123 (2)
S1—N1—H1N	115 (2)	S2—N2—H2N	113 (2)
C2—C1—C6	119.2 (3)	C14—C9—C10	118.3 (4)
C2—C1—S1	123.2 (3)	C14—C9—S2	117.4 (3)
C6—C1—S1	117.5 (3)	C10—C9—S2	124.0 (3)
C3—C2—C1	120.0 (3)	C11—C10—C9	120.7 (3)
C3—C2—Cl1	117.7 (3)	C11—C10—Cl2	118.1 (3)
C1—C2—Cl1	122.3 (3)	C9—C10—Cl2	121.2 (3)
C2—C3—C4	120.2 (4)	C12—C11—C10	120.1 (4)
C2—C3—H3	119.9	C12—C11—H11	120.0
C4—C3—H3	119.9	C10—C11—H11	120.0
C5—C4—C3	120.1 (3)	C11—C12—C13	119.9 (4)
C5—C4—H4	120.0	C11—C12—H12	120.0
C3—C4—H4	120.0	C13—C12—H12	120.0
C4—C5—C6	120.1 (4)	C12—C13—C14	120.8 (4)
C4—C5—H5	120.0	C12—C13—H13	119.6
C6—C5—H5	120.0	C14—C13—H13	119.6
C5—C6—C1	120.4 (3)	C13—C14—C9	120.1 (4)
C5—C6—H6	119.8	C13—C14—H14	119.9
C1—C6—H6	119.8	C9—C14—H14	119.9
O3—C7—N1	121.4 (3)	O6—C15—N2	120.6 (3)
O3—C7—C8	124.4 (4)	O6—C15—C16	124.6 (3)
N1—C7—C8	114.1 (3)	N2—C15—C16	114.7 (3)
C7—C8—H8A	109.5	C15—C16—H16A	109.5
C7—C8—H8B	109.5	C15—C16—H16B	109.5
H8A—C8—H8B	109.5	H16A—C16—H16B	109.5
C7—C8—H8C	109.5	C15—C16—H16C	109.5
H8A—C8—H8C	109.5	H16A—C16—H16C	109.5
H8B—C8—H8C	109.5	H16B—C16—H16C	109.5
			
O1—S1—N1—C7	42.9 (3)	O5—S2—N2—C15	−54.0 (3)
O2—S1—N1—C7	172.5 (2)	O4—S2—N2—C15	176.4 (3)
C1—S1—N1—C7	−71.7 (3)	C9—S2—N2—C15	61.2 (3)
O1—S1—C1—C2	172.8 (3)	O5—S2—C9—C14	−13.5 (3)
O2—S1—C1—C2	41.5 (3)	O4—S2—C9—C14	118.8 (3)
N1—S1—C1—C2	−71.0 (3)	N2—S2—C9—C14	−129.8 (3)
O1—S1—C1—C6	−5.5 (3)	O5—S2—C9—C10	172.2 (3)
O2—S1—C1—C6	−136.8 (3)	O4—S2—C9—C10	−55.6 (3)
N1—S1—C1—C6	110.7 (3)	N2—S2—C9—C10	55.9 (3)
C6—C1—C2—C3	0.3 (5)	C14—C9—C10—C11	0.2 (5)
S1—C1—C2—C3	−178.0 (3)	S2—C9—C10—C11	174.5 (3)
C6—C1—C2—Cl1	−180.0 (3)	C14—C9—C10—Cl2	178.7 (2)
S1—C1—C2—Cl1	1.8 (5)	S2—C9—C10—Cl2	−6.9 (4)
C1—C2—C3—C4	0.8 (6)	C9—C10—C11—C12	−0.5 (6)
Cl1—C2—C3—C4	−179.0 (3)	Cl2—C10—C11—C12	−179.1 (3)
C2—C3—C4—C5	−1.4 (6)	C10—C11—C12—C13	0.9 (6)
C3—C4—C5—C6	1.0 (6)	C11—C12—C13—C14	−0.9 (6)
C4—C5—C6—C1	0.1 (6)	C12—C13—C14—C9	0.7 (6)
C2—C1—C6—C5	−0.7 (5)	C10—C9—C14—C13	−0.3 (5)
S1—C1—C6—C5	177.7 (3)	S2—C9—C14—C13	−175.0 (3)
S1—N1—C7—O3	6.9 (4)	S2—N2—C15—O6	0.7 (5)
S1—N1—C7—C8	−173.2 (2)	S2—N2—C15—C16	−178.8 (3)

### Hydrogen-bond geometry (Å, °)

**Table:**

*D*—H···*A*	*D*—H	H···*A*	*D*···*A*	*D*—H···*A*
N1—H1N···O3^i^	0.85 (2)	2.03 (2)	2.848 (4)	162 (3)
N2—H2N···O6^ii^	0.84 (2)	1.96 (2)	2.788 (4)	172 (3)

Symmetry codes: (i) −*x*+1/2, *y*−1/2, −*z*+1/2; (ii) −*x*+3/2, *y*−1/2, −*z*+1/2.
